# Medical Colleges

**Published:** 1868

**Authors:** 


### EDITORIAL AND MISCELLANEOUS.

### MEDICAL COLLEGES.

Medical Colleges throughout the country seem to be active in their efforts to make more perfect their system of instruction. That which seems to impress all as most practically useful to students, is the amount and variety of clinical material. Institutions which, in the days of our pupilage, afforded literally nothing of the kind, now make "Clinical Medicine" an important item in the curriculum of study, and have daily instruction given in this department.

An increase in the number of Chairs beyond that of Clinical Medicine, and the extension of the term to six months, are being attempted by some Colleges, but few, we predict, will find students willing to remain during the protracted course, or able to digest the increased number of lectures in an ordinary term.

While we think that the system of instruction pursued by most Colleges is sadly deficient in many respects, and should be modified, and that the standard of qualification for the Degree is deplorably low, it is not very likely that the changes proposed in the mode of teaching will insure more thorough preparation of the student. That, and that alone, which will insure more rigid exactions in the green-room, will insure qualification.

When all are determined to effect this great subject, losing sight of all selfish considerations, "the way is easy." In 1866, our views on this subject were fully stated; and while we have no desire to press them, our opinion is not at all changed by the various propositions proposed for reform.

We are in receipt of announcement for the next course of lectures from the following Colleges:

*University of Virginia—Medical Department.*—In this Institution the course will open in October and continue *nine months.* The Medical Faculty admit students to examination for the Degree at the termination of one course. The Faculty consists of *five Professors.* Fees for the Course of Lectures, or Professor's tickets, $110.

*Bellevue Hospital College, New York.*—The regular Winter Course opens about the middle of October, and continues *four and a half months*—has *twelve Professors.* Fees for the course of lectures, $140. Graduates of last session, 111.

*Humbolt Medical College, St. Louis*, has a course of *seven months*, commencing the middle of September—has *eleven Professors.* Fees for the course of lectures, $105. Graduates last term, 4.

*University of Michigan, Ann Arbor.*—This Institution holds a session of *six months*, commencing the first of October—has *four Professors*, whose fees for the course is about $35.

*Jefferson Medical College, Philadelphia*, holds a course of *four and a half months*, with *seven Professors.* Fees for the course of lectures, $140. Graduates last term numbered 159, the largest of any school from which we have heard.

*Washington University, Medical Department, Baltimore, Md.*, opens the course of *four and three-fourths months* on the first of October, with *ten Professors.* Fees for the course of lectures, $120. Number of graduates last session, 55.

*Long Island College Hospital, Brooklyn, N. Y.*, commenced its regular Summer Course of *four months* on the first of March, with *nine Professors.* Fees for the course of lectures, $140. Number of graduates last term, 33.

*Willamette University, Medical Department, Salem, Oregon*, commences the course of *four months* on the fourth day of November—has *nine Professors*, with fees amounting to $110. At last term, graduated nine students.

*Miami Medical College, Cincinnati, Ohio*, opens on the first of October a course of *five months*, with *nine Professors.* Fees for the course of lectures, $60. Graduates at the last term, 30.

*University of Pennsylvania, Philadelphia*, commences its term of *four and a half months* in October, with a corps of *seven Professors.* Fees for the course of lectures, $140. Graduates at last term, 155.

*Albany Medical College, Albany, N. Y.*—This Institution opens on the first Tuesday in September, and continues *four months*, with *eight Professors.*

*University of Louisiana, New Orleans*, opens on the 16th of November, and continues *four months*, with *seven Professors.* Fees for the course of lectures, $140.

*University of Maryland, Baltimore, Md.*, opens its course of *four and a half months* on the 15th of October, with *seven Professors.* Fees for the course of lectures, $105.

*Toland Medical College, San Francisco, Cal.*, commences the regular course of *four months* in July, with *eight Professors.* Fees for the course of lectures, $130.

*The Medical College of Virginia, Richmond, Va.*, opens the regular course on the first of October, and continues *five months*, with *nine Professors.* Fees for the course of lectures, $120.

*Atlanta Medical College, Atlanta, Ga.*, opens, on the first of May, the regular course of *four months*, with *eight Professors.* Fees for the course of lectures, $120.

*University of Louisville, Medical Department, Louisville, Ky.*.—This Institution opens on the first of October, and continues its course *five months*, with *seven Professors* and several Assistants. Graduates, 24. Fees for the course of lectures, $40.

From the above, it will be seen that a large majority of the Colleges, whose announcement for the present year we have on file, have not found it practicable to make any considerable change in the number of Professors or length of the course. From the announcement for 1867 of six other Colleges, whose catalogues for the present year have failed to reach us up to this time, we see that five-sixths of them, at their last course, adhered to about the usual term and number of Professors. So that out of twenty-three Colleges, so far as we are informed, seventeen have from seven to nine Professors, and the term of lectures four to four and a half months. Of the six that have varied considerably from what has been usual, two have less than six Professors each, as will be seen in the above systematic notice of the different Colleges.

Highly as we appreciate all efforts to raise the standard of qualification for the Degree of Doctor of Medicine, and to improve the system of medical instruction, the plan proposed—that of increasing the number of Professors to ten or twelve and lengthening the term to six months—does not strike us as likely to be successful, nor, by any means, universally practicable, at this time.

By all means, lectures should be given upon all the subjects connected with medical science, and continued to the extent found useful; but more than this proves a source of embarrassment to both teacher and pupil. If two courses of lectures are not sufficient to prepare the student for examination—sufficiently close to protect the profession against the odium of having ignorant members—let the applicant be sent from the green-room to a third course, should he ask for it prematurely. Had the energies of the profession—particularly of those holding the positions of teacher and journalist—been so actively directed toward the discovery of some means of insuring rigid exactions in examination for the Degree, as in arranging plans for cramming pupils without their consent, the object would have been attained long since.

